# Sensitive Silver-Enhanced Microplate Apta-Enzyme Assay of Sb^3+^ Ions in Drinking and Natural Waters

**DOI:** 10.3390/molecules28196973

**Published:** 2023-10-07

**Authors:** Nadezhda S. Komova, Kseniya V. Serebrennikova, Anna N. Berlina, Anatoly V. Zherdev, Boris B. Dzantiev

**Affiliations:** A.N. Bach Institute of Biochemistry, Research Center of Biotechnology of the Russian Academy of Sciences, Leninsky Prospect 33, 119071 Moscow, Russia; nad4883@yandex.ru (N.S.K.); ksenijasereb@mail.ru (K.V.S.); zherdev@inbi.ras.ru (A.V.Z.); dzantiev@inbi.ras.ru (B.B.D.)

**Keywords:** antimony ions, microplate apta-enzyme assay, aptamer, oligonucleotide, adenine, thymine, water analysis

## Abstract

The toxic effects of antimony pose risks to human health. Therefore, simple analytical techniques for its widescale monitoring in water sources are in demand. In this study, a sensitive microplate apta-enzyme assay for Sb^3+^ detection was developed. The biotinylated aptamer A_10_ was hybridized with its complementary biotinylated oligonucleotide T_10_ and then immobilized on the surface of polysterene microplate wells. Streptavidin labeled with horseradish peroxidase (HRP) bound to the biotin of a complementary complex and transformed the 3,3′,5,5′-tetramethylbenzidine substrate, generating an optical signal. Sb^3+^ presenting in the sample bounded to an A_10_ aptamer, thus releasing T_10_, preventing streptavidin-HRP binding and, as a result, reducing the optical signal. This effect allowed for the detection of Sb^3+^ with a working range from 0.09 to 2.3 µg/mL and detection limit of 42 ng/mL. It was established that the presence of Ag^+^ at the stage of A_10_/T_10_ complex formation promoted dehybridization of the aptamer A_10_ and the formation of the A_10_/Sb^3+^ complex. The working range of the Ag^+^-enhanced microplate apta-enzyme assay for Sb^3+^ was determined to be 8–135 ng/mL, with a detection limit of 1.9 ng/mL. The proposed enhanced approach demonstrated excellent selectivity against other cations/anions, and its practical applicability was confirmed through an analysis of drinking and spring water samples with recoveries of Sb^3+^ in the range of 109.0–126.2% and 99.6–106.1%, respectively.

## 1. Introduction

At present, environmental pollution by heavy metals and metalloids is an extremely important problem. Antimony, as one of most dangerous pollutants of the biosphere, poses a significant problem due to its contamination of the environment, especially water bodies [1]. Antimony has trivalent and pentavalent states; however, the toxicity and poisonousness of Sb(III) is much higher [2]. The main sources of antimony causing its widespread pollution are the production of semiconductors and batteries, textiles, glass and ceramics and the action of coal-fired power plants and metal mining enterprises [3]. Another way Sb^3+^ enters into the human body is its release from polyethylene terephthalate packages, whose production requires antimony (III) oxide [4,5]. Upon entry to the human body through the respiratory tract, skin contact or the food chain, antimony degrades protein and carbohydrate metabolism and damages the liver, heart and nervous system [6], which confirms the demand for monitoring drinking water to assess the content of heavy metal ions [7], in particular antimony. In this regard, the maximum permissible concentration of total antimony in drinking water is 6 ng/mL, as established by the U.S. Environmental Protection Agency (EPA) [8], but the World Health Organization set the maximum permissible level of antimony in drinking water at 20 ng/mL [9].

The analytical techniques used for Sb^3+^ detection are atomic absorption spectrometry [10], inductively coupled plasma with mass spectrometry [11,12] and electrochemical analysis [13]. Although these methods are characterized by high sensitivity and selectivity, the need for expensive sophisticated equipment and long-term preparation of samples restricts their application. Therefore, simple and sensitive techniques for Sb^3+^ detection are an important area of development.

Currently, colorimetric methods deserve special attention as they offer a visual assessment of color change, allowing an easy readout of analytical signals. Colorimetric techniques for Sb^3+^ detection using indicator dyes [14,15,16] and nanoparticles [17,18,19] were developed. The application of dyes such as bis [2-(5-chloro-2-pyridylazo)-5-diethylaminophenolato]cobalt(III), bromopyrogallol red and pyrene causes the formation of complexes with Sb^3+^ and is followed by photo- or fluorometric detection. However, despite the simplicity and cost-effectiveness of these methods, they have insufficient selectivity and reproducibility and cannot ensure the detection of Sb^3+^ concentrations at the MPC level. Nanoparticle-based colorimetric methods are more usable, but they are sensitive to interfering components of matrixes [20]. Alternatively, the colorimetric method based on aptamers, due to their unique properties, can significantly expand the possibilities of determining heavy metal ions. Aptamers are short single-stranded oligonucleotides that are characterized by high affinity and specificity for their specific analytes, and their low production cost makes aptamers relevant analytical reagents [21,22]. Aptamer-based assays have confirmed effectiveness for the detection of heavy metal ions, as summarized in the reviews [23,24].

In particular, a homogeneous colorimetric technique for the detection of Sb^3+^ based on the aggregation of aptamer-modified gold nanoparticles, accompanied by a rapid color transition from pink to blue, was developed [19]. Aptamer A_10_ was used as the receptor molecule, forming a complex with Sb^3+^ due to interaction with hydroxyl groups of adenine residues [25,26]. However, the detection limit of this assay was 10 ng/mL, which does not reach the established MPC levels. In this regard, an important and urgent task is to find new and better analysis formats that provide the required level of detectable concentrations. One such method is a simple microplate format of an assay for highly sensitive determination. Some microplate methods using aptamers and nanozymes have been previously reported and developed for the determination of various analytes [27,28]. The application of aptamers in these methods provides high specificity and reproducibility, as well as excellent storage stability, and the use of nanozymes allows for increased assay sensitivity. Thus, these advantages have been used to develop approaches for the analysis of the commonest heavy metal ions [29,30,31]. Several microplate colorimetric aptamer-based methods have been developed, including one for the detection of cadmium using gold-nanoparticle-modified MoS_2_ [29] and one for detecting lead ions, involving graphene/Fe_3_O_4_-AuNP composites and the recognition element [31], and rolling circle amplification as a signal amplifier has been proposed for mercury ion detection [30]. However, to the best of our knowledge, the creation of a microplate aptamer-based assay for the determination of Sb^3+^ ions has not previously been reported.

In this study, a microplate apta-enzyme assay for Sb^3+^ detection with a low detection limit is reported for the first time. The proposed microplate method is based on the destruction of a complex of complementary aptamers due to the affinity of aptamer A_10_ toward antimony ions. Compared with the nanozyme-based approaches described above, this assay is implemented using simple and readily available materials and combines the widely used horseradish peroxidase as a label and specific aptamer recognition. To reach high sensitivity, the assay conditions were varied, including the amount of immobilized biotinylated complementary complex, concentration of streptavidin and incubation time. In addition, the effect of Ag^+^ on the analytical performance of Sb^3+^ detection was investigated. The ability of the developed assay to selectively distinguish Sb^3+^ from other cations and anions and its practical applicability for drinking and spring water sample testing were studied.

## 2. Results and Discussion

### 2.1. Principle of Sb^3+^ Detection

**Figure 1:**
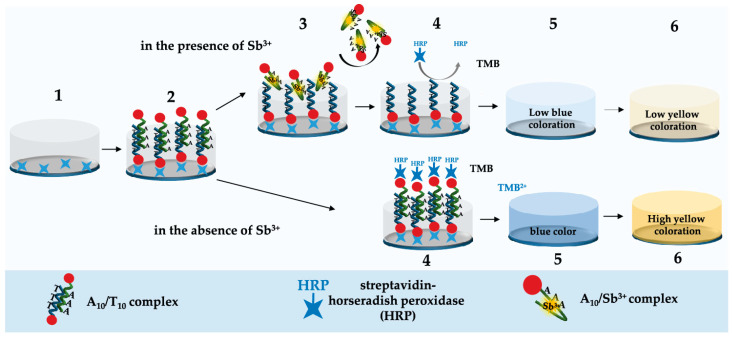
Schematic representation of microplate apta-enzyme assay for Sb^3+^ detection. (1)—sorption of streptavidin, (2)—binding of the A_10_/T_10_ complex, (3)—binding of Sb^3+^, (4)—binding of streptavidin labeled with horseradish peroxidase, (5)—addition of TMB, (6)—stopping oxidation by addition of H_2_SO_4_.

**Figure 2:**
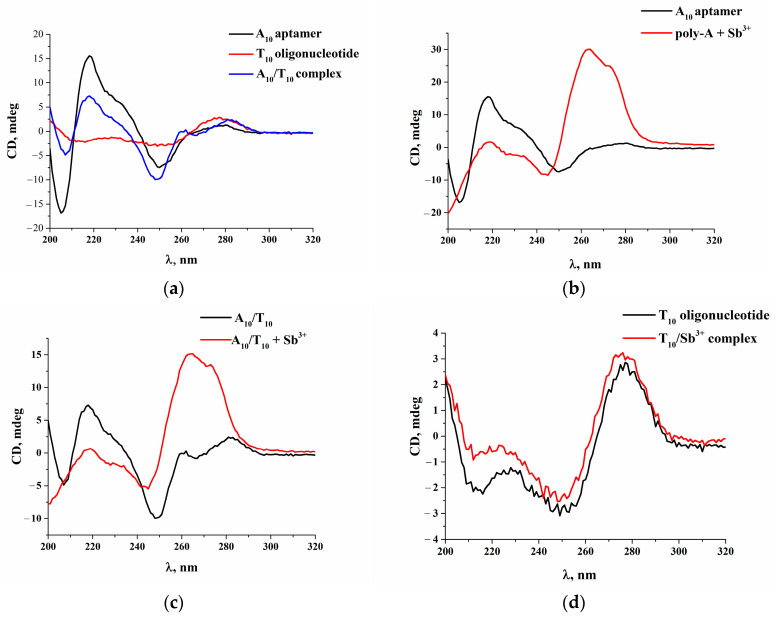
(**a**) CD spectra of A_10_ aptamer, T_10_ oligonucleotide and A_10_/T_10_ complex; (**b**) CD spectra of A_10_ aptamer before and after addition of Sb^3+^; (**c**) CD spectra of A_10_/T_10_ complex before and after addition of Sb^3+^; (**d**) CD spectra of T_10_ oligonucleotide before and after addition of Sb^3+^. Concentrations of A_10_ and T_10_ were 500 µg/mL. Concentration of Sb^3+^ was 100 µg/mL.

The scheme of the designed microplate apta-enzyme assay using Sb^3+^-specific aptamer A_10_ and complementary oligonucleotide T_10_ is shown on Figure 1. In this assay, the aptamer A_10_ and complementary T_10_ oligonucleotide, both biotinylated at the 5′ end, were used. Firstly, streptavidin was adsorbed in the microplate wells (Figure 1, 1), and then the preliminary formed complementary biotinylated A_10_/T_10_ complex was immobilized (Figure 1, 2) through high-affinity biotin–streptavidin interaction. The binding of the A_10_/T_10_ complex to the immobilized streptavidin was made possible by the biotinylated end of A_10_ or T_10_, and both variants stored the Sb^3+^ binding ability of the A_10_ aptamer. Next, incubation with HRP-streptavidin (Figure 1, 4) led to its binding to the free biotin of the A_10_/T_10_ complex. At the next stage, the addition of TMB substrate promoted the development of blue color (Figure 1, 5), which turned yellow after stopping the reaction with sulfuric acid (Figure 1, 6).

The presence of Sb^3+^ led to a switching of the A_10_ aptamer structure, resulting in the formation of the A_10_/Sb^3+^ complex. At the same time, the bonds of the immobilized A_10_/T_10_ complex were broken [19,25]. In the case of immobilization of the A_10_/T_10_ complex via the biotinylated end of T_10_, the resulting A_10_/Sb^3+^ complex was removed after washing the microplate. If the A_10_/T_10_ complex was immobilized via the biotinylated end of A_10_, the formed A_10_/Sb^3+^ complex remained on the microplate, and T_10_ was removed during washing. In both cases, washing the microplates reduced the number of HRP-streptavidin binding sites, causing a decrease in the following transformation of the TMB substrate and the registered intensity of the blue color.

### 2.2. Characterization of the Apta-Assay Components via CD Spectroscopy

The CD spectrum of the aptamer A_10_ had a large positive peak at 270 nm and a negative minimum at 248 nm (Figure 2a, black line), which is consistent with previous findings [32,33]. Thus, the conformation of the single-stranded oligonucleotide was characterized by a large positive band in the range of 260–270 nm due to base stacking and a negative band at 240 nm due to polynucleotide helicity [34,35]. The CD spectrum of the oligonucleotide T_10_ had a positive peak near 275 nm and a less intense band at 227 nm (Figure 2a, red line). The positive peaks at 282 and 260 nm and a negative peak at 247 nm (Figure 2a, blue line) confirmed the formation of the A_10_/T_10_ complex, which accords with the spectrum of the B-form conformation of the double-stranded oligonucleotide [36]. To confirm the formation of the A_10_/Sb^3+^ complex, the spectra of the A_10_ aptamer (Figure 2b), the T_10_ oligonucleotide (Figure 2d) and the A_10_/T_10_ complex (Figure 2c) with Sb^3+^ were recorded. The intensity of the positive band at 218 nm decreased after the addition of Sb^3+^ to the A_10_ aptamer, and the intensity of the band at 275 nm increased significantly (Figure 2b), similarly to the change in the CD spectrum for the A_10_/T_10_ complex with Sb^3+^ (Figure 2c). The interaction of Sb^3+^ with the T_10_ oligonucleotide was not associated with spectral changes (Figure 2d). The results of CD spectroscopy confirm the feasibility of the proposed assay and demonstrate its applicability for the detection of Sb^3+^.

### 2.3. Optimization of the Microplate Apta-Enzyme Assay Conditions

**Figure 3:**
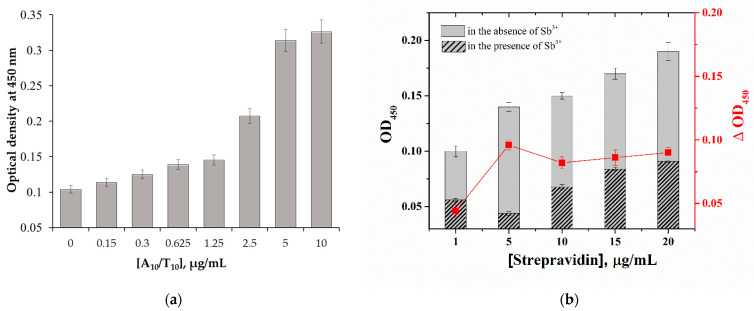
(**a**) Influence of the A_10_/T_10_ complex concentration on the registered OD_450_ in the proposed assay. Experimental conditions were 10 μg/mL streptavidin, no Sb^3+^. (**b**) Influence of streptavidin concentration on the registered OD_450_. Experimental conditions were 5 μg/mL A_10_/T_10_ complex, 10 µg/mL of Sb^3+^. The curve shows the difference between OD_450_ in the absence and in the presence of Sb^3+^. Error bars show the standard deviations for three replicate measurements.

To optimize the conditions of the microplate apta-enzyme assay for sensitive Sb^3+^ detection, the concentrations of streptavidin and the A_10_/T_10_ complex as well as incubation time were varied. First, the effect of the concentration of the A_10_/T_10_ complex on the analytical signal in the absence of Sb^3+^ was investigated. According to Figure 3a, as the concentration of the A_10_/T_10_ complex increased up to 5 µg/mL, the optical density at 450 nm (OD_450_) gradually grew. After this, OD_450_ no longer increased, which indicates the saturation value for the A_10_/T_10_ complex. Therefore, 5 μg/mL was chosen as the optimal concentration of the A_10_/T_10_ complex. In addition, consistent immobilization of the biotinylated A_10_ aptamer (2.5 μg/mL) and biotinylated T_10_ oligonucleotide (2.5 μg/mL) to assemble the A_10_/T_10_ complex into the microplate wells was tested. However, it was found that the immobilization of the pre-formed complex showed better binding of A_10_/T_10_ and reduced analysis time due to the absence of an additional step.

At the next stage, the effect of the concentration of streptavidin, which provides the required number of binding sites for the A_10_/T_10_ complex, was investigated (Figure 3b). The streptavidin concentration of 5 μg/mL showed the highest difference between OD_450_ values in the absence of Sb^3+^ and in its presence at a fixed concentration, 10 µg/mL. The ΔOD_450_ data for a concentration of 5 μg/mL were compared with the data for 1 and 10 μg/mL using Student’s *t*-test, which confirmed the significance of differences (*p* < 0.05). In addition, the comparison of detection limits of Sb^3+^ showed the lowest value (42 ng/mL) for 5 μg/mL immobilized streptavidin, which was chosen as the optimal concentration.

Finally, the incubation time of Sb^3+^ solutions was studied, which influenced the disruption of the A_10_/T_10_ complex and the formation of the A_10_/Sb^3+^ complex. The OD_450_ reached a plateau at 45 min, and this value was selected as an optimal time for incubation of the Sb^3+^ solution.

As a result, the optimal conditions for the microplate apta-enzyme assay were defined as follows: 5 μg/mL streptavidin, 5 μg/mL A_10_/T_10_ complex and 45 min of incubation time for Sb^3+^.

### 2.4. Analytical Performance of the Microplate Apta-Enzyme Assay of Sb^3+^

**Figure 4:**
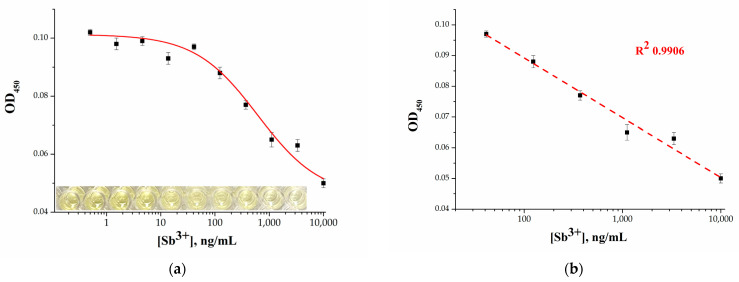
(**a**) Dependence of the optical density on Sb^3+^ concentration. Inset: photo of microplate wells for the same concentrations of Sb^3+^; (**b**) linear range of the calibration curve for Sb^3+^ detection. Error bars show the standard deviation for three replicate measurements.

Under the optimal conditions, solutions with different concentrations of Sb^3+^ were tested (Figure 4a). The color intensity reached the maximum in the absence of Sb^3+^, and the color of solution was bright yellow. With an increase in the concentration of Sb^3+^, the intensity of the color weakened accordingly. However, the presence of a background signal, even at high concentrations of Sb^3+^, was observed, which is possibly due to the nonspecific effect of the biotinylated T_10_ oligonucleotide remaining on the microplate wells. The optical densities at 450 nm in response to Sb^3+^ concentrations varying from 0.5 ng/mL to 10 µg/mL are shown in Figure 4a. A linear relationship (Figure 4b) between the OD_450_ value and the logarithmic concentrations of Sb^3+^ was acquired, which corresponded to 40–10,000 ng/mL with a correlation coefficient R^2^ = 0.9906. The detection limit was calculated as the blank signal (in the absence of Sb^3+^) minus 3σ (where σ is the standard deviation of the signal) and determined to be 42 ng/mL, which is higher than the MPCs recommended for drinking water.

### 2.5. Ag^+^-Enhanced Microplate Apta-Enzyme Assay for Sb^3+^ Detection

Since the obtained detection limit did not reach the maximum permissible concentration for Sb^3+^ in drinking water, the other metal ions were considered as enhancing agents to provide high sensitivity. It is known that the introduction of metal ions into duplexes of oligonucleotides leads to new properties of the formed metal–aptamer systems. Thus, it was found that the addition of Ag^+^ ions to the A_10_/T_10_ complex led to the formation of a metallo-DNA system without changes in the canonical hybridization of the Watson–Crick duplex [37]. At the same time, the affinity of adenine–thymine binding was reduced [38], which thereby promoted the dehybridization of the A_10_ aptamer and more efficient formation of the A_10_/Sb^3+^ complex.

**Figure 5:**
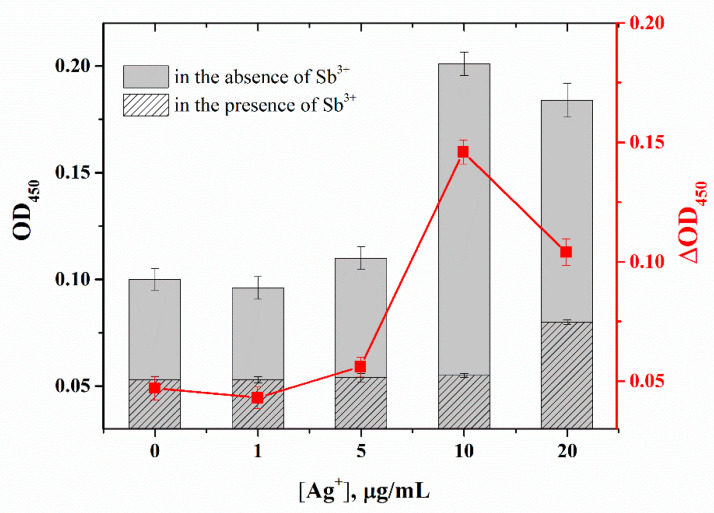
Effect of Ag^+^ concentration on optical density for the proposed microplate apta-enzyme assay. Experimental conditions were 5 μg/mL A_10_/T_10_ complex, 10 μg/mL streptavidin. The curve shows the difference between OD_450_ in the absence and in the presence of Sb^3+^. Error bars show the standard deviation for three replicate measurements.

First, the effect of Ag^+^ and Sb^3+^ on the catalytic activity of horseradish peroxidase was tested to exclude artifacts that may change the assay results. The absence of the inhibition of this process in the presence of these metal ions was shown. To determine the optimal amount of Ag^+^, the addition of different concentrations of Ag^+^ was tested and detection limits of Sb^3+^ were calculated (Figure 5). The most pronounced increase in the detection limit of Sb^3+^ was observed at a Ag^+^ concentration of 10 µg/mL, which was chosen as the optimal concentration of the enhancing agent. The significance of the increase in the analytical signal at a concentration of 10 μg/mL was compared with the values at 5 and 20 μg/mL using Student’s *t*-test, which confirmed the significance of the deviation (*p* < 0.05).

**Figure 6:**
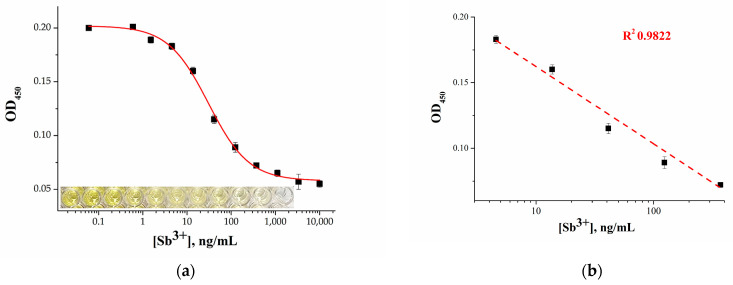
(**a**) Calibration curve for Sb^3+^ detection using the Ag^+^-enhanced microplate apta-enzyme assay. Inset: photo of microplate wells for the same concentrations of Sb^3+^. (**b**) Linear range of the calibration curve. Error bars show the standard deviation for three replicate measurements.

The calibration curve for the Ag^+^-enhanced microplate apta-enzyme assay was plotted in the range of 0.5 ng/mL–10 µg/mL (Figure 6a). A linear relationship (Figure 6b) between OD_450_ and the logarithmic concentrations of Sb^3+^ was acquired, which corresponded to 13.7–1111 ng/mL and was regressed to y = 0.22 − 0.058x (R^2^ = 0.9822).The detection limit for the enhanced assay was determined to be 1.9 ng/mL, which is 22 times lower than that of the previous variant. In addition, the achieved detection limit turned out to be 3–10 times lower than the recommended values of MPC for drinking water established by international organizations.

### 2.6. Selectivity of Ag^+^-Enhanced Microplate Apta-Enzyme Assay

**Figure 7:**
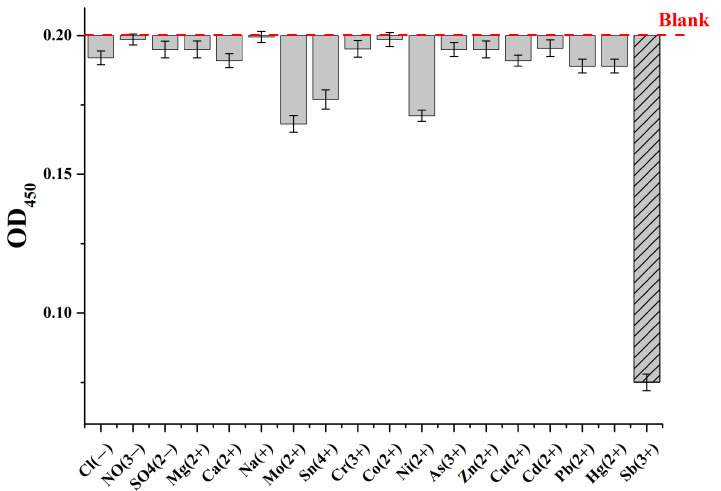
Selectivity of the developed Ag^+^-enhanced microplate apta-enzyme assay for Sb^3+^ ions concerning other cations and anions. The concentration of each ion was 100 ng/mL. Error bars show the standard deviation for three replicate measurements.

The selectivity of the Ag^+^-enhanced microplate apta-enzyme assay of Sb^3+^ was investigated. The target ion was compared with other toxic cations, such as Pb^2+^, Hg^2+^, Cd^2+^, Cu^2+^, Zn^2+^, As^3+^, Ni^2+^, Co^2+^, Cr^3+^, Sn^4^ and Mo^2+^. In addition, cations (Ca^2+^, Na^+^, Mg^2+^) and anions (Cl^−^, NO^3−^, SO_4_^2−^) presenting in high amounts in water were tested using the developed assay. As follows from Figure 7, the results show that most of the ions tested did not have a noticeable effect on the determination of Sb^3+^. However, insignificant nonspecific changes were observed in the presence of Ni^2+^, Sn^4+^ and Mo^2+^ ions, which may have an effect on the determination of Sb^3+^ ions. The effect of these ions was observed at 0.1 μg/mL, while the allowable concentration of nickel and molybdenum ions in drinking water is 0.07 and 0.01 μg/mL, respectively. In the case of Sn^4+^, its presence in this form is practically impossible and, in addition, the Sn^4+^ content in drinking water is not standardized. Thus, the proposed system showed satisfactory selectivity with respect to antimony ions.

### 2.7. Water Sample Analysis

The effectiveness of the developed Ag^+^-enhanced assay was studied via analysis of drinking and spring water samples. Since the results of the standard ICP-MS reference method do not show the presence of the analyte, the water samples were spiked with different concentrations of Sb^3+^. The calculated analytical recoveries were within acceptable ranges of 109–126% and 99.6–112% for drinking and spring water, respectively (Table 1), which is consistent with published results for the determination of heavy metals in analytical practice [39,40,41]. Thus, the development of a colorimetric assay [41] based on the regulation of the oxidase-mimicking activity of Mn_3_O_4_ nanoparticles by oligonucleotides for mercury and cadmium ions in actual environmental water samples achieved a detection rate in the range of 85.7 to 105.21% for mercury ions and 81.7 to 113.4% for cadmium ions. The work [40] demonstrates a mean recovery of samples in the range of 94.6–124% via an ultrasensitive aptamer-based biosensor for arsenic(III) detection. Thus, the results indicate the potential application of the Ag^+^-enhanced microplate apta-enzyme assay for Sb^3+^ detection in water samples.

### 2.8. Comparison with Other Methods

The comparison of different methods for Sb^3+^ detection in Table 2 demonstrates the advantages of the developed Ag^+^-enhanced microplate apta-enzyme assay in relation to both instrumental and colorimetric methods. The LOD of the proposed method (1.9 ng/mL) is comparable to that of flame atomic absorption (emission) spectrometry [42,43], inductively coupled plasma mass spectrometry [12,44], excimer fluorescence [16] and surface-enhanced Raman scattering [45,46], which require the use of expensive equipment. In addition, the proposed method demonstrates more advantageous sensitivity compared to the very small number of low-tech methods available for the determination of Sb^3+^ [19,47].

## 3. Materials and Methods

### 3.1. Chemicals and Materials

The aptamer 5′-biotin-AA-AAA-AAA-AA-3′ (A_10_) and the complementary oligonucleotide 5′-biotin-TT-TTT-TTT-TT-3′ (T_10_) were synthesized by Syntol (Moscow, Russia). Aqueous solutions of Sb^3+^, Ag^+^, Hg^2+^, Pb^2+^, Cd^2+^, Cu^2+^, Zn^2+^, As^3+^, Ni^2+^, Co^2+^, Cr^3+^, Sn^4+^, Mo^2+^, Cl^-^ and SO_4_^2-^ were purchased from the Center of Standardization of Samples and High-Purity Substances (Saint Petersburg, Russia). The streptavidin and horseradish-peroxidase-labeled streptavidin (HRP-Streptavidin) were from IMTEK (Moscow, Russia). The bovine serum albumin and Tween-20 were obtained from Sigma-Aldrich (St. Louis, MO, USA). The 3,3′,5,5′-tetramethyl benzidine (TMB), sulfuric acid and salts for buffer solutions were purchased from Chimmed (Moscow, Russia). All solutions were prepared using the Simplicity Water Purification System from Millipore (Bedford, MA, USA). The 96-well transparent polystyrene high bind 9018 plates were obtained from Corning Costar (Corning, NY, USA).

### 3.2. Formation of A_10_/T_10_ and A_10_/Ag^+^/T_10_ Complexes

The biotinylated A_10_ aptamer and T_10_ oligonucleotide were dissolved at the final concentration of 5 µg/mL in 20 mM Tris-HCl buffer containing 150 mM NaCl, 1 mM MgCl_2_ and 1 mM CaCl_2_. To form a complementary aptamer complex, the reaction mixture was heated at 95 °C for 5 min and kept at room temperature (RT) for 15 min.

To obtain the A_10_/Ag^+^/T_10_ complex, Ag^+^ solution at the final concentration of 10 µg/mL was added to the mixture of A_10_ aptamer and T_10_ oligonucleotide (2.5 µg/mL for both compounds) in the same buffer solution, followed by heating at 95 °C for 5 min and cooling to RT for 15 min.

### 3.3. Circular Dichroism Measurement

Circular dichroism (CD) spectra were recorded using the CD spectrometer Chirascan from Applied Photophysics (Leatherhead, UK). The CD spectra were collected at RT in the range of 200–320 nm with 3 repetitions. The investigated aptamer and the complementary complex were preliminary prepared in 20 mM Tris-HCl buffer containing 150 mM NaCl, 1 mM MgCl_2_ and 1 mM CaCl_2_, annealed at 95 °C for 5 min and cooled down to RT.

### 3.4. Microplate Apta-Enzyme Assay

A total of 100 μL of streptavidin at a concentration of 5.0 μg/mL in 50 mM phosphate-buffered saline, pH 7.4 (PBS), was applied to the wells of the microplate and incubated overnight at 4 °C. Then, the microplate was washed three times with 10 mM PBS containing 0.05% detergent Tween-20 (PBST). The microplate wells were covered with 150 μL of 1% BSA (blocking buffer) prepared in 50 mM PBS for 30 min at 37 °C in a thermostat TC-1/80 (Russia). After three washes with PBST, 100 μL of freshly prepared A_10_/T_10_ complementary complex, 5 µg/mL in 20 mM Tris-HCl buffer, pH 7.6, was added in the wells. The microplates were incubated for 1 h at 37 °C and then washed with PBST three times. At the next stage, 100 μL of solutions containing different concentrations of Sb^3+^ were added into the wells. After 1 h of incubation at 37 °C and triple washing, 100 μL of horseradish-peroxidase-labeled streptavidin (1:5000 dilution in PBST) was added to the wells and incubated again for 1 h at 37 °C. The unbound HRP-labeled streptavidin was removed and washed three times with PBST and once with distilled water. The final steps were the addition of 100 mL of TMB substrate solution and incubation for 15 min at RT. The catalytic reaction was stopped by the addition of 50 μL of 1 M H_2_SO_4_ per well. Then, the optical density at 450 nm as a colored product of peroxidase-induced oxidation of TMB was measured using an EnSpire multimode plate reader from PerkinElmer (USA). The plotting of (optical density—Sb^3+^ concentration) dependence was carried out using Origin 9 software (OriginLab Corporation, Northampton, MA, USA).

The Ag^+^-enhanced microplate apta-enzyme assay was carried out using the A_10_/Ag^+^/T_10_ complex, as described above.

### 3.5. Preparation and Analysis of Water Samples

Samples of drinking and spring water with the pH adjusted to 8.5 were filtered through a syringe filter with a pore size of 0.2 µm (Sartorius, Germany). According to ICP-MS data (quadrupole mass spectrometer Nexion 300D, Perkin Elmer (Waltham, MA, USA)), the used water samples were reliably free of Sb^3+^. The method’s description is provided in [49]. The results of the water analysis using the ICP-MS technique have been published in our previous works on heavy metal detection; see supplementary information in [49,50]. A series of Sb^3+^-spiked water samples were prepared at concentrations of 10, 50 and 100 ng/mL with the addition of a Sb^3+^ standard solution (0.1 mg/mL in the Milli-Q water). Then, spiked water samples were analyzed with the developed microplate apta-enzyme assay.

## 4. Conclusions

A sensitive microplate apta-enzyme assay for Sb^3+^ detection was proposed for the first time. The main principle of this approach is based on the formation of a specific complex of the A_10_ aptamer with Sb^3+^, leading to a reduction in the recorded transformation of the TMB substrate. It was found that Ag^+^ promoted dehybridization of the A_10_ complex of the complementary oligonucleotide and the formation of the A_10_/Sb^3+^ complex. Under optimized conditions, the results show that the analytical performance was enhanced by up to 22 times in the presence of Ag^+^. The achieved detection limit, 1.9 ng/mL, is 3–10 times lower than the established MPC values. The main benefits of the developed assay are its (i) low detection limit; (ii) cost-efficiency and (iii) simplicity. Additionally, this assay relies on available materials, such as conjugates of streptavidin with horseradish peroxidase and combinations of biotinylated aptamers. Finally, the proposed assay was applied in spiked drinking water samples with satisfactory results.

## Figures and Tables

**Table 1:** Detection of Sb^3+^ in water samples using Ag^+^-enhanced microplate apta-enzyme assay (n = 3).

Samples	Added, ng/mL	Found, ng/mL	Recovery, %
Drinking water	10.0	10.9 ± 0.3	109.0 ± 2.8
	50.0	63.1 ± 0.2	126.2 ± 0.3
	100	115.0 ± 0.2	115.0 ± 0.2
Spring water	10.0	11.2 ± 0.3	112.0 ± 2.7
	50.0	49.8 ± 0.7	99.6 ± 1.4
	100	106.1 ± 1.1	106.1 ± 1.0

**Table 2:** Parameters of the detection of Sb^3+^ for various methods.

Method	LOD, ng/mL	Probe	Ref.
Sophisticated Instrumental Methods			
Inductively coupled plasma–optical emission spectrometry	24.9–32.3	water, basal culture medium, anaerobic sludge plus basal medium	[44]
Ion-assisted photochemical vapor generation with inductively coupled plasma mass spectrometry	0.0047	lake and river water	[12]
Bulk optode coupled with spectrophotometry using 6-(4-(2,4-dihydroxyphenyl)diazenyl)phenyl)−2-oxo-4-phenyl-1,2-dihydro pyridine-3-carbo-nitrileas a ionophore	0.85	tap, domestic, sea, ground, lake water, blood plasma, urine	[48]
Surface-enhanced Raman scattering using silvered porous silicon and phenylfluorone	1	-	[45]
T-shaped slotted quartz tube–atom trap–flame atomic absorption spectrometry	0.75	mineral water	[42]
Excimer fluorescence using pyrene as a sensing probe	160	-	[16]
Hydride generation coupled with atmospheric pressure glow discharge atomic emission spectrometry	0.14	groundwater	[43]
Surface-enhanced Raman spectroscopy using dithiothreitol-functionalized two-dimensional Au@Ag array	1	natural water	[46]
Low-tech methods			
Colorimetric method based on the development of a yellow potassium iodoantimonite complex	600	water samples from mine adits	[47]
Colorimetric detection using gold nanoparticles modified with poly-adenine aptamer	10	drinking water	[19]
Ag^+^-enhanced microplate apta-enzyme assay using poly-adenine aptamer	1.9	drinking and spring water	This work

## Data Availability

The data that support the findings of this study are available from the corresponding author upon request.
